# Domestic violence against women and associated factors in Ethiopia; systematic review

**DOI:** 10.1186/s12978-015-0072-1

**Published:** 2015-08-29

**Authors:** Agumasie Semahegn, Bezatu Mengistie

**Affiliations:** College of Health and Medical Sciences, Haramaya University, Po. Box- 235, Harar, Ethiopia

## Abstract

**Background:**

Violence against women is now widely recognized as a serious human right abuse, and an important public health problem with substantial consequences physical, mental, sexual, and reproductive health. Data on systematic review of domestic violence are needed to support policy and program recommendations. Therefore, the overall purpose of this systematic review was to assess magnitude of domestic violence against women and associated factors in Ethiopia.

**Methods:**

Studies systematically reviewed in Federal Democratic Republic of Ethiopia from 2000 to 2014. Systematic review was employed on published research works from databases such as Pubmed, popline, Hinari, and Google using key words. We also consulted public health experts. Community based studies with a study population (15–49 years) were included for review. Thirteen peer reviewed papers and two consecutive Ethiopian demographic and health surveys (2005 and 2011) were included to the systematic review. Twenty seven available in open access journals were retrieved and assessed based on the criteria’s such as community based study, cross sectional study design, clearly report prevalence and associated factors were included in the systematic review work. Finally, 15 papers were included in this review.

**Results:**

Lifetime prevalence of domestic violence against women by husband or intimate partner among 10 studies ranged from 20 to 78 %. The lifetime domestic physical violence by husband or intimate partner against women ranged from 31 to 76.5 %. The life time domestic sexual violence against women by husband or intimate partner ranged from 19.2 to 59 %. The mean life time prevalence of domestic emotional violence was 51.7 %. Significant number of women experienced violence during their pregnancy period. Domestic violence against women significantly associated with alcohol consumption, chat chewing, family history of violence, occupation, religion, educational status, residence and decision making power.

**Conclusion:**

Domestic violence against women was relatively high in different parts of Ethiopia. Domestic violence has direct relationship with sociodemographic characteristics of the victim as well as perpetrator. Therefore, appropriate health promotion information activities needed to tackle associated factors of domestic violence against women or to prevent and control the problem to save women from being victim.

## Introduction

Violence is defined by the world health organization (WHO) as intentional use of physical force or power, threatened or actual, against oneself, another person, against a group or community that either results in or has a high likelihood of resulting in injury, death, psychological harm, mal development or deprivation [1]. Domestic violence against women is universal phenomenon that persists in all countries of the world and a major contributor of ill health of women. The perpetrators are often well known to their victims [2]. The health social, sexual, reproductive health and wellbeing of millions of individuals and families is adversely affected by violence [1, 3, 4]. Domestic violence is now widely recognized as serious human rights abuse, and increasingly as an important public health problem with substantial consequences for women’s physical, mental, sexual, and reproductive health [5]. The health system often are not adequately addressing the problem of violence and contributing to comprehensive multi-sectoral response [1, 3].

Worldwide, 40–70 % of female murder by their intimate partner. No country in the world is women safe from violence. According WHO multicountry study, domestic violence ranged from 15 % in Japan to 71 % in rural Ethiopia [2, 6–8]. Domestic violence has gained prominence around the world as grave violation of human and legal rights. Women are usually the victim of domestic violence that derives from unequal power relationships between men and women [7]. More than three women murder per day by their husbands in the United States. About 1,181 women murder by their intimate partner in 2005. About 2 million women experience injuries from intimate partner violence each year. About 84 % of women are victim of spouse abuse. Women of all ages are at risk of domestic violence [8].

Domestic violence against women results physical, sexual, mental harm or suffering to women, including threats, coercion or arbitrary deprivation of liberty occurring in public or in private life [9]. Violence in the domestic sphere usually perpetrate by husband/intimate partner. It often occurs in life cycle. About 20 to 50 % women experience domestic violence worldwide. Women’s successful campaigning raise the profile of the issue of Violence against women (US conferences; Vienna, 1993; Cairo, 1994; and Beijing, 1995) recognize women’s rights as an indisputable part of universal human rights [10, 11]. The center of disease control estimates the costs of domestic violence in the United States alone exceed US$5.8 billion per year: US$4.1 billion for direct medical and health care services; productivity losses US$1.8 billion [6, 8].

Domestic violence against women is major obstacle on progress on achieving development targets. Without addressing it, anybody have little chance of meeting millennium development goals [7, 11]. Domestic violence continues to have an unjustifiably low priority on the international development agenda, planning, programming and budgeting [11]. Domestic violence links with wide range of reproductive health issues such as sexual transmitted infections including HIV, miscarriages, risky sexual health behaviour [8]. Domestic violence against women has strong link with HIV/AIDS. Women living with HIV more likely experience violence and woman who experiences violence more likely acquire HIV either direct risk of infection or creating an environment unable to adequately protect themselves [12].

Domestic violence against women occurs in all social and economic classes, but women living in poverty more likely to experience violence. More research required to fully understand the connections between poverty and domestic violence against women [11]. Women are victim of domestic violence at a rate about 5 times that of males. In US, domestic violence is most prominent among women aged 16 to 24. Poorer women experience significantly more domestic violence than higher income women [6, 8]. Domestic violence is common in Ethiopia both urban and rural families. About 68–81 % of women agree wife beating if husband has justify in at least one of specified situations in Ethiopia [13]. About 88 % of rural and 69 % of urban women believe that their husbands have the right to beat them. Approximately, one out of ten women do victim of abduction, early forced marriage, rape and marital rape. Marital rape is still not recognized under the criminal code 2005. Ethiopia government revises family law in 2000 and criminal law in 2005 to protect women right. The criminal code and constitution article 35(4) condemn harmful traditional practices. Ethiopia ratified the convention on the elimination of all forms of discrimination against women in 1981 [13, 14]. However, there is a paucity of country wide evidence about domestic violence against women and associated factors in Ethiopia.

Since this systematic review conducted on studies done in different parts of Ethiopia in which 84 % of population lives in rural and poor resource settings. The finding from different studies would close the information gap regarding the current situation of domestic violence against women and helps for government officials, policy makers and any other concerned bodies to design prevention and controlling strategies to alleviate. Therefore, this systematic review aimed to determine prevalence of domestic violence against women and its associated factors in Ethiopia.

### Conceptual framework



## Methods

### Study setting and period

This systemic review was conducted from October to November 2014 on studies conducted in Ethiopia from 2000 to 2014. Ethiopia is bordered by Eritrea to the North and North East, Djibouti and Somalia to the East, Sudan and South Sudan to the West, and Kenya to the south. Ethiopia is the second populous (94.1 million) country in Africa with total area of 1,100,000 km^2^. Addis Ababa is the capital city. The oldest evidence for modern humans is found in Ethiopia which is widely considered the region from which Homo sapiens. Ethiopia is a multilingual society more than 80 ethnic groups. Oromo and Amhara are the two largest ethnic groups.

### Searching strategy

Systematic review was employed through defining domestic violence against women and associated factors in Ethiopia. Published research works searched using computerised search engines such as Pubmed, popline, Hinari, direct Google and consulted public health experts. Published papers from Ethiopia were identified. All of the published papers entitled domestic violence against women, intimate partner violence against women, violence against women and associated factors in Ethiopia were considered for systematic review. Community based study, cross sectional study design, population (15–49 years old women), published from 2000 to 2014, studies different parts of Ethiopia and perprators being husband or intimate partner were used to select papers for review. Published papers searched from internet using keywords, and phrases such as “sexual violence against women in Ethiopia”, “physical violence against women in Ethiopia”, “emotional violence against women in Ethiopia”, “intimate partner violence against women in Ethiopia”. Only published papers included to the systematic review except Ethiopian demographic and health survey report. The demographic and health survey finding included because of population based survey which has been conducted in Ethiopia. The title and the abstract critically reviewed after downloading the papers. Some of the papers excluded here. In the second phase full document thoroughly read and reread to include the paper for review. Finally, only fifteen studies from 2000 to 2014 in different parts of Ethiopia met the inclusion criteria to the systematic review to assess the prevalence of domestic violence against women (15–49) and associated factors.

### Criteria for inclusion of studies

Published articles identified by assessing the title and abstract for relevance to the review objective, and then retrieved the full text for further assessment. Those articles clearly report the prevalence of different types of domestic violence (sexual, physical, psychological or emotional) and associated factors using adjusted odd ratio as well as have high response rate (greater than 95 %) consider as high quality articles. The quality of the articles assessed by the two authors through setting criteria’s such as sample size, aim of the study, measured variables, study design, definition of the outcome variable, the method used to assess the domestic violence. All papers published before 2000 were excluded from the systematic review. Based on the criteria articles study design, number of participant, objective of the study and major findings such as magnitude of sexual, physical, emotional violence against women by their husband/intimate partner and associated factors using 95 % CI and adjusted odds ratio were extracted. Therefore, all articles which used community based study, cross sectional study design and clearly report the prevalence and associated factors were included.

### Definition of exposure and outcome

Comparison of studies for systematic reviews challenged by variety of ways that has been defined domestic violence against women by husband or intimate partner. In the majority of the studies included in this systematic review to assess domestic violence against women were assessed using the their demographic and health surveys and WHO multi country study domestic violence assessment tool by contextualizing or modifying to their study objective or local contexts. Sexual violence’s such as forced sex without the consent of the woman, having sex when women do not want, having unusual type of sex that hurts her. Physical violence (beating): any form of violent act which can result in physical harm including slapping, punching, kicking, beating with any object, twisting the arms, strangulation, using knife or gun against women. Psychological or emotional abuse includes physical intimidation, threats of abandonment, uttering humiliating things (insulting) confinement to home and withholding money. These variables were asked to the women using different question, for example “Has he slapped, kick you, drag or beaten you?” “have you ever been insulted by your husband using abusive language that made you feel bad about yourself?” “have you ever physically forced by your husband to have sex when you did not want to?” etc. The main outcome considered in this review was domestic violence (such as sexual, physical, emotional/psychological). Domestic violence against women mean the women’s had experienced at least one form among the three by their husband/intimate partner.

## Results

### Description of studies

**Fig. 1:**
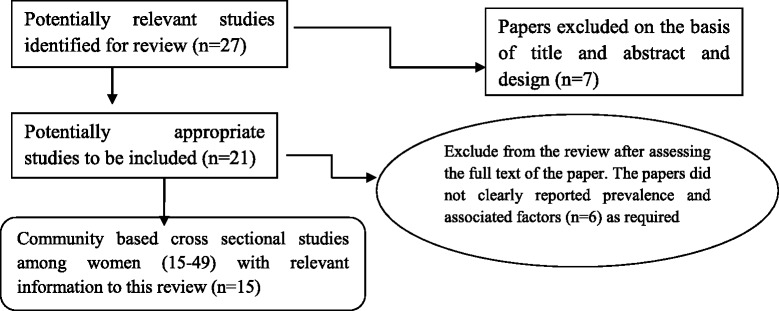
The flow chart that show how to select papers for systematic review

Fifteen peer reviewed papers used on the analysis and interpretation of data. All the information from selected papers was described their prevalence and significantly associated factor findings. Findings of selected papers were described and presented on tables constructed in Microsoft word. The corresponding author name, study area, study objectives, design of the study, total number of participant (n), main finding [prevalence and associated factors] were clearly labeled. Variables that showed significant statistical association with domestic violence against women by husband/intimate partner using 95 % CI of the adjusted odd were reported (Fig. 1).

### Magnitude

There are limited number of study has been conducted on domestic violence against women in Ethiopia. Fifteen published papers found in different parts of Ethiopia from 2000 to 2014 from international and national journals. Main finding of the fifteen studies found in Table 1. In addition, demographic and health survey 2005 and 2011 findings were included in this systematic review. Almost all of the studies were used community based cross sectional study design which composed of urban and rural resident women. However, there were two journals which are qualitative and institution (refuge) studies. The socio-demographic characteristic of women and perpetrator resident (urban or rural), age, education, decision making, family history of violence, occupational status were considered as contributing factor and analyzed on most of the reviewed studies. Nevertheless, very few of the studies were involved both men and women interview particularly on attitude and perception of community assessment. All of the studies included women in the reproductive age (15–49).

**Table 1:** Domestic violence against women and associated factors studies from different parts of Ethiopia; 2000-November, 2014

Author	Study area	Objective	Population sampling	Main finding (domestic violence)
Yigzaw T. et al., 2004 [15]	Gondar Zuria district, which is one of the 16 districts of the North Gondar Administrative Zone in northwest Ethiopia	Aimed to assess the prevalence of domestic violence and associated factors of intimate partner violence	1104 women (15–49) who selected using systematic sampling	Women who ever experienced physical, sexual, and/or psychological abuse were 50.8 %. The prevalence of physical violence was 32.2 %, while that of forced sex and physical intimidation amounted were 19.2 % and 35.7 %, respectively. Exposure to parental domestic violence [AOR = 12.9; 95 % CI 9.1, 18.5] as girl was the strongest risk factor for being victim of violence later in life while husband used alcohol was the major attribute of violent partners [AOR = 4.7; 95 % CI 3.13, 6.9]. Rape was more likely to occur among rural women [AOR = 1.9;95 % CI: 1.3, 3.0], women who witnessed parental violence [AOR = 3.8; 95 % CI% 2.7, 5.4] and spouses consume alcohol frequently [AOR;2.9; 95 % CI 1.9, 4.2] while rape was less likely [AOR:0.7; 95 % CI 0.5, 0.9] to occur among women who shared household decision making.
Semahegn et al. 2013 [16]	FagitalekomaWoreda	Aimed to determine magnitude of domestic violence and identify its predictors	682 Married women (15–49) and systematic sampling	The prevalence of domestic violence was 78.0 %. About 73.3 %, 58.4 % and 49.1 % of women reported different forms of psychological, physical and sexual violence, respectively. Husband alcohol use [AOR = 1.9, 95%CI: 1.3, 2.8], being pregnant [AOR = 2.1, 95 % CI: 1.4, 3.4], decision making power [AOR = 2.3, 95 % CI: 1.5, 3.4] and annual income [AOR = 1.9, 95 % CI: 1.1, 3.3] were predictors of domestic violence.
Hassen and Deyassa, 2013 [17]	7districts in South Wollo zone	Aimed to assess the relationship between sexual violence and HIV infection among clients of VCT services	647 women (15–49) who selected	The prevalence of lifetime partner sexual violence, and last 12 months partner sexual violence were 34.6 %, and 10.5 % respectively. The overall prevalence of HIV among VCT users was 21.5 %. The chance of having HIV was 1.97 times higher among women victims who have a history of lifetime partner violence when compared with women who are not victims; [COR = 1.97, 95 % CI: 1.34 - 2.90].
NegussieDeyessa et al.,2010 [18]	Butajira district	To examines the contribution of area of residence and literacy to rates of violence against women, focusing on norms and attitudes of women in Ethiopia.	A total of 3,016 women (15–49) who selected by simple random sampling	Te over all physical violence against women during the last 12 months was 32 %. Fewer of these women were in polygamous marriages. Literate women living in rural communities had the highest prevalence of experiencing physical violence [AOR_2.2; 95 % CI: 1.3_3.7]. Similarly, spousal literacy alone, more women living with a literate spouse in rural communities had experienced physical violence [AOR_1.7; 95 % CI: 1.1_2.5]. Rural women had significantly higher odds of experiencing physical violence [AOR_3.4; 95 % CI: 1.7, 6.9].
Annabel Erulkar,2013 [19]	The survey took place 31 districts from 7 regions in Ethiopia	Aimed to explore the relationship Early Marriage, Marital Relations and Intimate Partner Violence in Ethiopia	1,671 women aged 20–24 who selected using simple random sampling method	Only one in five young women attained some secondary education. About 30 % got married at ages 15–17. Married women (18–19) were more likely than those married before age 15 to have discussed sexual issues [24 % vs. 15 %; p < 0.01]. who had married before age 15 were less likely to have wanted to experience sexual initiation than who had married at ages 18–19 [49 % vs. 85 %; p < 0.001], Likewise, the youngest brides had experienced high levels of forced first sex with their husbands (32 %) were more likely than older brides [7 % vs. 2–3 %; p < 0.001].
Feseha et al. 2012 [20]	Shimelba refugee camp, Northern Ethiopia	aim to assess the magnitude of intimate partner physical violence and associated factors among women in Shimelba refugee camp, Northern Ethiopia	422 refugee women (15–49) who selected by simple random sampling method	The prevalence of physical violence in the last 12 months and lifetime were 25.5 % and 31.0 % respectively. The commonest forms of physical violence reported slapping (61.6 %) and throwing objects (19.5 %). Physical violence associated with being farmer [AOR; 3.0: 95% CI: 1.7, 5.5], knowing women in neighborhood whose husband to beat them [AOR; 1.87 95% CI: 1.0, 3.5], being Muslim [AOR;2.4: 95%CI: 1.1, 5.5], and having husband drunk hard [AOR;2.1:95% CI:1.0, 4.5]. Women whose husband drink alcohol, chew khat or smoke cigarette experience IPV were higher than their counter parts [AOR;1.9: 95% CI: 1.2, 3.0] and [AOR;3.6: 95%C.I: 2.0, 6.2] respectively.
WHO, 2005 [1]	rural Meskan and Mareko District	To investigate how such violence is associated with ill-health and injury, and the strategies that women use to cope with the violence	3016 women between 15–49 years of age	Nearly one half (49 %) of women experienced physical violence by partner at some point in their lives, and 29 % during the past 12 months. 59 % of women experienced sexual violence at some point, and 44 % during the past 12 months. About 71 % of women experienced one or more form of violence over their lifetime. About 35 % of all women experienced at least one severe form of physical violence. Ninety eight percent of women experienced physical violence during pregnancy period by their husband. Of these, 28 % of pregnant women had been punched/kicked on abdomen. But 39 % of the women had kept silent
Deribe K, Beyene BK, Tolla A, Memiah P, Biadgilign S, et al. (2012) [21]	Kersa and Sokoru districts, Jimma Zone of Oromiya region southwest Ethiopia	To assess the magnitude of intimate partner violence in Southwest Ethiopia in predominantly rural community	851 married Women (15–49) who selected using Systematic sampling technique	The life time prevalence of sexual or physical violence or both was 64.7 % (95%CI: 61.4 %–67.9 %). The lifetime sexual violence [50.1 %; 95 % CI: 46.7 %–53.4 %] was considerably higher than physical violence [41.1 % (95 %:37.8–44.5)]. Women reported physical/sexual violence, or both, in the past year [41.5 %;95 % CI: 38.2 %–44.8 %]. Women who did not believe wife could do anything if husband wants were more likely to report physical violence [AOR = 3.4; 95 % CI: 1.5–7.6]. Woman with a controlling partner were more likely report physical violence [AOR = 6.4; 95% CI: 3.8–10.8). Housewives were less likely report sexual violence than employed women [AOR = 0.6; 95%CI: 0.4–0.9].
Amare Deribew, 2007 [22]	Agaro, Jimma Zone, south western Ethiopia	To assess the magnitude, type and risk factors of intimate partner violence against women in Agaro town, Southwest Ethiopia.	510 women (15–49) who selected by systematic sampling	The lifetime prevalence of husband/intimate partner violence was 51.8 %. About 32 %, 33 % and 46 % of women had physical, sexual and emotional abuses in their lifetime, respectively. Majority of the physical (80.9 %) and emotional (80.7 %) abuses occurred in the last year. The common acts of physical violence were slapping (68.7 %), pushing (62.0 %) and hitting with fist/stick (27 %). About 28 % of them experienced severe form of physical violence such as hitting with fist, choking and threatening with gun. Sexual and physical violence were more than 2 times likely occur among women whose partner consumed alcohol more frequently [OR = 2.3; 95 %: 1.43, 3.54].
W. Shanko, 2013 [23]	Kersa district, Oromia region Ethiopia	Aimed to assess the knowledge and prevalence of domestic violence among women in Kersa district of Oromia region.	858 women (15–49) who selected by systematic sampling method	Only 39.7 % of women reported that they recognized that domestic violence against women was a problem in their area. Ever experience of domestic violence against women was significantly related to Amhara ethnicity and age group 30–49 years. Only 19.9 % women who ever experienced violence reported it to the legal authorities.
Abeya et al. 2011 [24]	one urban (Nekemte) and 4 rural districts in East Wollega Zone	Aimed to investigate the prevalence, patterns and associated factors of intimate partner violence against women in Western Ethiopia	1540 ever married who selected systematic random sampling	Lifetime and past 12 months prevalence of intimate intimate/husband violence against women was 76.5 % [95 % CI: 74.4-78.6 %] and 72.5 % [95 % CI: 70.3-74.7 %], respectively. The overlap of psychological, physical, and sexual violence was 56.9 %. Rural residents [AOR 0.6, 95 % CI 0.3-0.98], literates (AOR 0.7, 95 % CI 0.5-0.9), female headed households (AOR 0.5, 95 % CI 0.3-0.8). Older women were nearly four times [AOR 3.4, 95 % CI 1.2 -8.9]. Abduction (AOR 3.7, 95 % CI 1.0-13.6), polygamy (AOR 3.8, 95 % CI 1.6-0.7), Husband alcohol use [AOR 2.0 95 % CI 1.2- 3.2], and previous witnesses of parental violence [AOR 2.00, 95 % CI 1.5-2.6] factors associated with an increased likelihood of lifetime IPV.
Abeya et al. 2012 [25]	one urban (Nekemte) and 4 rural districts in East Wollega Zone	aimed to explore the attitudes of the community on intimate partner violence against women	On 60 women were selected for Discussants purposefully	Most discussants perceived, IPV is accepted in the community in circumstances of practicing extra marital sex and suspected infidelity. The majority of women are keeping silent and very few defend themselves from the violent husbands/partners. The suggested measures by the community to stop or reduce women’s violence were targeting actions at the level of individual, family, community and society
EDHS, 2011 [13]	Country wide survey	To assess the demographic and health condition in the country	More than 17,000 households	Domestic violence is common in Ethiopia, in both urban and rural families. About 68 % agreed that wife beating is justified in at least one of the specified situations. Women with no education are more than three times as likely as women with more than secondary education to agree with at least one specified justification for wife beating (79 % and 22 %, respectively). Similarly, 81 % of women in the lowest wealth quintile agree with at least one specified justification for wife beating versus 46 % of women in the highest wealth quintile.
EDHS, 2005 [26]	Country wide	To assess the demographic and health condition in the country	14,500 households from 540 clusters was selected	Majority of women (81 %) believe that a husband is justified in beating his wife for at least one of the specified reasons. About 86 % of rural women agree with at least one of the reasons justifying wife beating, compared with 59 % among urban woman
Deyessa et a, 2009 [27]	Two districts called Meskan and Mareko in Guraghe zone	The aim of the present study is to examine the relation between IPV and depression in a community-based study in Butajira	A total of 1994women (15–49) who were selected by simple random sampling	The lifetime prevalence of husband/intimate partner violence was 72.0 %[95 % CI: 70.0 %-73.9 %]. Lifetime prevalence of intimate partner physical violence was 49.5 % [95 % CI: 47.4 %-51.7 %]. Physical violence [OR = 2.6, 95 % CI, 1.6, 4.1], childhood sexual abuse [OR:2.0, 95 % CI, 1.1-3.6], mild emotional violence [OR:3.2, 95 % CI,2.0-5.1], severe emotional violence [OR:3.9, 95 % CI,2.2-6.9) and high spousal control of women [OR:3.30, 95 % CI: 1.6-6.9] associated with depressive episode.

*AOR* means adjusted odds ration; *COR* mean crude odds ratio

Finding from review of the cross sectional community based studies showed that more than half (50.5 %, 78 %, 72 %, 64.7 %) of women experienced domestic violence (includes physical, sexual and psychological), respectively [15, 16, 21, 27]. The life time domestic violence against women by husband or intimate partner in Ethiopia which ranged 19.2–78.0 % (mean value of 60.6 %). The life time physical violence by husband or intimate partner against women or wives ranged from 31 to 76.5 % (mean value of 47.7 %) in different parts of Ethiopia [1, 15, 16, 18, 20–24, 27]. The life time sexual violence against women by husband or intimate partner in Ethiopia ranged from 19.2 to 59 % (with mean value of 39.6 %). Of these, one in five women experienced having forced sexual intercourse by their husband/intimate partner [1, 15–17, 19, 21, 22]. Similarly one in five women experienced forced sex and experienced violence during their pregnancy period [1, 15, 16, 20]. The life time prevalence of emotional violence was 51.7 % [15, 16, 21]. Approximately three quarter of women experienced repeated beating such as hit by sticks, slapped, kicked on different parts of their bodies, punched, stabbed and different harassment mechanisms. More than one third (35.7 %) of women reported threats of battering, threatened to damp, endured verbal degradation, deprived the freedom to go out, withheld money and other family support [15]. Similarly one fourth of women had experienced both physical and sexual violence [21]. Another cross sectional community based study in Tigray and South Wollo zone showed that prevalence of lifetime sexual violence by an intimate partner was 32.3 % [95 % CI: 28.7–35.9 %]. Almost half of women experienced physical violence during their lifetime by their husband or intimate partner [17, 20]. More than a quarter of the women experienced moderate or severe forms of emotional violence, and more than half were partially or completely restricted in what they could do, requiring permission from their spouse. Women who had experienced were significantly associated with being victim of intimate partner violence (Physical, sexual and emotional violence) women had experienced intimate partner violence and victim of physical injuries during their pregnancy period. [27] [Table 1].

### Factors associated with domestic violence

Most of studies reported that domestic violence significantly associated with husband alcohol consumption, khat chewing, family history of violence, partner education, decision making power, residence (rural women more victim of violence), women age less than 18 at first sex or marriage makes victim of violence. Women literacy negatively associated with domestic violence that increases the risk of violence, having extra partner, religion in rare situations [15–20]. Similarly, women experienced any form of intimate partner violence associated significantly with age group, lower educational status, khat chewing and the woman's occupational status [22, 27]. Two factors associated with physical violence, Those women who did not believe a wife could do anything if a husband wants a girlfriend were more likely to report physical violence [AOR:3.4; 95 % CI: 1.5–7.6]. Woman with a controlling partner were more likely to report physical violence (AOR = 6.4; 95 % CI: 3.8–10.8). Similarly two factors associated with sexual violence. Housewives were less likely report sexual violence than working women [AOR; 0.6; 95 % CI: 0.4–0.9]. Concurrences with physical violence womenwith controlling partner were more likely report sexual violence than their counterpart [AOR = 4.7; 95 % CI: 2.8–7.9] [21] [Table 1]. In Ethiopia, Housewives more likely kept silent due to considered it as part of family life, show of love and economic dependency on the husband.

Eight out of ten women accept wife beating if the husband/intimate partner have at least one justifiable reason. women and men were asked whether husband is justified hitting or beating his wife in each of the following five situations: if she burns the food; if she argues with him; if she goes out without telling him; if she neglects the children; and if she refuses to have sexual relations with him [13, 22]. Among all respondents, 124(18.2 %) women reported the presence of traditional gender norm that support wife beating. “....mostly…the women themselves accept wife beating by the husband…he is my husband and he can kick me…” one in twenty women accepting the traditional gender norm that support wife beating. Majority of women kept silent from seeking help. However, mostly report to their family, local elders, leaders and religious fathers [15, 16]. Almost four in ten (39 %) of physical violence victim women kept silent. However, few abused women asked formal agencies or authorities for help (such as local leader, health services, police and the courts) ranged from 1 to 85 %). Among those women who did not seek help, majority of women said they feared the consequences and 37 % said they considered the violence “normal” or “not serious”[1, 16]. One study conducted in Agaro south western Ethiopia reported that 27 % of husbands abuse their wives/partner without clear reasons. Other perceived triggering factors of violence included: women’s disobedience (27 %), problems/conflict in either of the partners’ family (26 %), problem faced by the male at work site (24 %), jealousy (14 %) and poverty/low income (13.5 %) [21].

## Discussion

This systematic review of studies determined the prevalence of domestic violence against women and associated factors in Ethiopia. Two third of women did experience domestic violence by their husband or intimate partner. Approximately half of women experienced physical violence as well and emotional violence by their husband/intimate partner. The mean life time prevalence of emotional violence was 51.7 %. Domestic violence significantly associated with substance abuse (alcohol consumption and chat chewing), family history of violence, occupation being housewife, religion being Muslim, educational being literate status, residence being rural and decision making power. Majority of the women kept silent without reporting the violence to concerning bodies that are in position or authority. Approximately three quarter of women accept wife beating if husband has at least one justified. Significant number of women had experienced domestic violence during their pregnancy period by the father of the child and victimized so many injuries.

This study finding of emotional violence is inlined with the systematic review in North America (47 %) which also had high levels of emotional violence (78 %) along with respondents studied in South America, Europe and Asia (37–50 %) and, for emotional violence, the highest rates but our focus is partner violence [28]. This is quite higher than the finding from systematic review from sub Saharan African countries showed that women who justified wife beating ranged from Madagascar (28 %) to Ethiopia (74 %) [29]. The difference might be due to the presence of traditional gender norm that support wife beating and women themselves accept wife beating. The finding from this review is almost similar with studies done in India and Bangladeshi revealed that 69.7 % and 52.1 % of women experienced domestic violence, respectively [30]. However, the prevalence of domestic violence against women in Ethiopia relatively higher than as compared with study conducted in Nepal, almost half of women reported violence in which one in five women reported sexual and physical violence. Approximately 45 % of women experienced physically forcing her to have sexual intercourse when she did not want it. More than one in ten had kicked, dragged or beaten [31]. This gap might be due to traditional gender norms that support women inferiority, and also significant number of women accept wife beating by their husband/intimate partner. This finding is quite higher than study rural Indonesia revealed that lifetime sexual and physical violence was 22 % and 11 %. Sexual violence was associated with husbands’ young age, educated less than 9 grades [32].

This is comparable with finding from studies in Sub-Saharan Africa that showed 13–49 % of women physically assaulted by husband. One province in Zimbabwe finds that 26 % (with 20 % and 40 %) of women have ever been forced to have sex while they did not unwanted. About 10 % in Zimbabwe and 7 % in South Africa of women physically assaulted during pregnancy [33]. On the other hand, almost half of women reported domestic violence in Zambia (48 %), Colombia (44 %), and Peru (42 %). In Egypt, Nicaragua, Cambodia, Dominican Republic and India about one in three married women reported the experience of domestic violence. Approximately 7 in 10 domestic violence victim women not sought any help from any bodies [16]. Similarly, WHO multicountry shows that 11 % in Nicaragua (11 %) and Colombia (1 %).of women reported spousal abuse during pregnancy. Many women did not seek help for the violence range from 41 % in Nicaragua to 78 % in Cambodia [34]. Still this study is higher than the systematic review and meta-analysis of 7 studies that lifetime partner violence was 45.8 % range 15.6–89.2 %. Domestic violence against women was 35.3 % (1.7–82.5 %) during past year [35]. This finding is two times higher than study done in rural area of Bangalore, in which 29.57 % of women reported domestic violence such as verbal abuse (81.6 %), physical abuse (31.6 %), psychological abuse (27.6 %) and sexual abuse (10.5 %) [36]. This finding is consistent with study in Nepal revealed that prevalence of domestic violence was 63.1 % (95 % CI 61.2–65.05). Male who had more than three or equal children were less likely to have perpetrated domestic violence compared with those who had less children [37]. No or little inter-spousal communication and low autonomy of women significantly increases the odds of experiencing violence [31]. Both couples with higher educated significantly lower likelihood of experiencing both types of violence (AOR; 0.9, CI: 0.9–0.9) and severe violence (AOR;0.9, CI: 0.8–0.9) than low educated [30].

It is consistent with a study done in Bangalore, India that 57 % of women reported experience of physical domestic violence. These women were married on average year longer (*p* <0.001) and were more likely to have children (*p* < 0.001) associated with women experience violence than others than. Over two thirds of women who had ever experienced domestic violence reported that their husbands had difficulty to find or keep job than their counter parts (*p* <0.001). Employed women during study visit had 60 % higher odds of violence by her subsequent visit than unemployed women (AOR = 1.6, 95 % CI: 1.1–2.3) [38]. The systematic result from studies in Ethiopia is higher than WHO multi-country finding in 2005 that 15–71 % of women had experienced physical or sexual violence, or both, at some point in their lives [39]. This result is almost similar with worldwide study showed that alcohol consumption associated with domestic violence against women by their husband /intimate partner. Childhood experiences of violence in the home reinforce for both men and women the normative nature of violence thus increasing the likelihood of male perpetration and women’s acceptance of abuse [40]. This finding is quite higher compared with study conducted in Malawi reported that 13 %, 20 %, 13 % of women reported emotional, physical, and sexual violence, respectively. Data showed women ages 15–19 were less likely report emotional violence. But women ages 25–29 were more likely report physical violence (OR 1.4; CI: 1.1–1.7), and women ages 30–34 were more likely report sexual violence, compared to women age 45–49 (OR 1.4; CI: 1.0-1.9) [41].

### Implication of the study

Domestic violence against women is still important public health problem. Although Ethiopia is state party to many international and regional human rights instruments including theconvention on the elimination of discrimination against women. Domestic Violence against women by their husband or intimate partner has different negative social, economical, emotional, Health, sexual and reproductive health outcomes or consequences. Without addressing violence against women could not achieving growth and development targets, in which it recognize as a public health andhuman rights concern in Ethiopia. Therefore, determining magnitude and associated factors from different reviews’can helpgovernment officials, policy makers, program designers and any concerned bodies to design prevention and controlling strategies to tackledomestic violence. Preventing violence against women has key role to the achievement of the eight MDGs that specifically addresses promotion of gender equality and women's empowerment (MDG-3). Information obtained here can be used forplanning of intervention programs in different part of the country.

### Strength and limitation of the study

This systematic review used community based cross sectional studies. Majorities of the journal are quantitative study that used to determine the magnitude and associated factor. However, very few studies were qualitative study that used to assess the community attitude and perception towards violence against women (wife beating). As limitation all studies used cross-sectional study design that has limitation to determine causality. This might induce social desirability bias during self reporting of the violence because of cultural barrier for disclosure sensitive and family secrets.

## Conclusion

Domestic violence against women was relatively high in different parts of Ethiopia. More than half of women experienced domestic violence against women by their husband or intimate partner at their home. The problem has direct relationship with different sociodemographic characteristics of the victim as well as perpetrator. Approximately three quarter of women accepted wife beating if husband has at least one justified reason. Therefore, we recommend that the government policy makers, program planners and other concerned bodies (nongovernmental organizations) to establish appropriate strategy to prevent and control violence against women. Prevent wife beating in the community by integrating programs on domestic violence with health extension program.

## References

[1] WHO. WHO Multi-country study on women’s’ health and domestic violence against women. World Health Organization, Geneva Switzerland. 2005. http://www.cih.uib.no/journals/EJHD/ejhd17-special-issue-2/ejhdv17-special-issue-2-2003-cover.htm or http://www.who.int/gender/violence/en/ accessed October 2014.

[2] WHO (2014). Strengthening the role of the health system in addressing violence, in particular against women and girls, and against children.

[3] UNFPA. Addressing Violence against Women and Girls In Sexual And Reproductive Health Services: A Review Of Knowledge Assets.

[4] World Bank. Ending Violence against Women and Girls Reduction and prevention of gender-based violence as a contribution to the protection of human rights and to development. Eschborn, Germany 2003. http://www.gtz.de accessed April 2013.

[5] Garcia-Moreno C, Jansen HAFM, Ellsberg M, Heise L, Watts CH (2006). Prevalence of intimate partner violence: fi ndings from the WHO multi-country study on women’s health and domestic violence. Lancet.

[6] UNIFEM, World Council of Churches, the World Student Christian Federation and the World YWCA.Amnesty USA. Domestic Violence: A Global Problem March 24 – March 30, 2010 Lenten Study 6. http://women.overcomingviolence.org. Accessed Nov 2014.

[7] Futures without violence. The Facts on Domestic, Dating and Sexual Violence. Formerly family violence prevention fund. www.futureswithoutviolence.org/resources-events/get-the-facts Accessed Nov 2014.

[8] USAID, Interagency Gender Working Group (LGWG). Gender-Based Violence: Impediment to Reproductive Health. Population Reference Bureau. 2010. www.igwg.org/igwg_media/gbv-impediment-to-RH.pdf. accessed November 2014.

[9] Violence against women. Fact sheet 239. Geneva, World Health Organization, 2009. (http://www.who.int/mediacentre/factsheets/ fs239/en/, accessed Nov 2014.

[10] UNICEF. domestic violence against women and girls. Innocenti Digest no. 6, june 2000. UNICEF Innocenti Research Centre, Piazza SS. Annunziata, 12 50122 Florence, Italy. www.unicef. accessed Nov 2014.

[11] WHO. Addressing violence against women and achieving the Millennium Development Goals. Geneva, Switzerland. World Health Organization 2005. (http://www.who.int/gender/en accessed Nov 2014.

[12] WHO, UNAIDS, UNFPA, Addressing violence against women and HIV/AIDS What works?World Health Organization, Geneva, Switzerland. 2010. www.who.int/reproductivehealth Accessed Nov 2014.

[13] Central Statistical Agency [Ethiopia] and ORC Macro (2012). Ethiopian demographic health survey 2011.

[14] Africa for women right ratify and respect. Women’s rights protection instruments ratified by Ethiopia. www.wikigender.org or www.africa4womensrights.org accessed Nov 2014.

[15] Yigzaw T, Yibrie A, Kebede Y (2004). Domestic violence around Gondar in northwest Ethiopia. EthiopJHealth Dev.

[16] Semahegn A, Belachew T, Abdulahi M (2013). Domestic violence and its predictors among married women in reproductive age in Fagitalekoma Woreda, Awi zone, Amhara regional state, North Western Ethiopia. Reprod Health.

[17] Hassen F, Deyessa N (2013). The relationship between sexual violence and human immunodeficiency virus infection among women using voluntary counseling and testing services in South Wollo Zone, Ethiopia. BMC Research Notes.

[18] Deyessa N, Berhane Y, Ellsberg M, Emmelin M, Kullgren G, Gberg HU (2010). Violence against women in relation to literacy and area of residence in Ethiopia. Global Health Action.

[19] Erulkar A (2013). Early marriage, marital relations and intimate partner violence in Ethiopia. Int Perspect Sex Reprod Health.

[20] Feseha G, G/mariam A, Gerbaba M (2012). Intimate partner physical violence among women in Shimelba refugee camp, northern Ethiopia. BMC Public Health.

[21] Deribe K, Beyene KB, Tolla A, Memiah P, Biadgilign S, Amberbir A (2012). Magnitude and correlates of intimate partner violence against women and its outcome in southwest Ethiopia. PLoS ONE.

[22] Deribew A (2007). Magnitude and risk factors of intimate partner violence against women in agaro town, southwest Ethiopia. Ethiopian J Health Sci.

[23] Shanko W, Wolday M, Assefa N, Aro AR (2013). Domestic violence against women in Kersa, Oromia region, eastern Ethiopia. East Mediterr Health J.

[24] Abeya SG, Fantahun MA, Worku AY (2011). Intimate partner violence against women in western Ethiopia: prevalence, patterns, and associated factors. BMC Public Health.

[25] Abeya SG, Fantahun MA, Worku AY (2012). Intimate partner violence against women in west Ethiopia: a qualitative study on attitudes, woman’s response, and suggested measures as perceived by community members. Reprod Health.

[26] Central Statistical Agency [Ethiopia] and ORC Macro (2006). Ethiopian demographic health survey 2005.

[27] Deyessa N, Berhane Y, Alem A, Ellsberg M, Emmelin M, Hogberg U, et al. Intimate partner violence and depression among women in rural Ethiopia: a cross-sectional study. Clinical Practice Epidemiology Mental Health. 2009;5(8):1–10. 10.1186/1745-0179-5-8 PMC2689215 19397834

[28] Alhabib S, Nur U, Roger J (2010). Domestic violence against women: systematic review of prevalence studies. J Fam Viol.

[29] Uthman OA, Lawoko S, Moradi T (2010). Sex disparities in attitudes towards intimate partner violence against women in sub-Saharan Africa: a socio-ecological analysis. BMC Public Health.

[30] Rapp D, BeateZoch MM, Khan H, Pollmann T, Krämer A (2012). Association between gap in spousal education and domestic violence in India and Bangladesh. BMC Public Health.

[31] Lamichhane P, Puri M, Tamang J, Dulal B (2011). Women’s status and violence against young married women in rural Nepal. BMC women?’. Health.

[32] Hayati EN, Högberg U, Hakimi M, Ellsberg MC, Emmelin M. Behind the silence of harmony: risk factors for physical and sexual violence among women in rural Indonesia. BMC Women?’. Health. 2011;11(52):1–8. 10.1186/1472-6874-11-52 PMC3257195 22112243

[33] Watts C, Mayhew S (2004). Reproductive health services and intimate partner violence: shaping a pragmatic response in Sub-Saharan africa. Int Fam Plan Perspect.

[34] ORC Macro. Profile of domestic violence on multi country study; MEASURE DHS+ surveys; Calverton, USA. http://www.measuredhs.com. Accesed October 2014.

[35] Trevillion K, Oram S, Feder G, Howard LM (2012). Experiences of domestic violence and mental disorders: a systematic review and meta-analysis. PLoS ONE.

[36] Gaikwad V, Madhukumar S, Sudeepa D (2011). An epidemiological study of domestic violence against women and its association with sexually transmitted infections in Bangalore rural. Online J Health Allied Scs.

[37] NandBhatta D (2014). Shadow of domestic violence and extramarital sex cohesive with spousal communication among males in Nepal. Reprod Health.

[38] Krishnan S, Rocca CH, Hubbard AE, Subbiah K, Jedmeades J, Padian SN (2010). Do changes in spousal employment status lead to domestic violence? Insights from a prospective study in Bangalore, India. Soc Sci Med.

[39] Mary E, Jansen HAFM, Lori H, Watts CH, Claudia G-M (2008). Intimate partner violence and women’s physical and mental health in the WHO multi-country study on women’s health and domestic violence: an observational study. Lancet.

[40] Rachel Jewkes. Intimate partner violence: causes and prevention. THE LANCET . 2002; 359: 1423–29. www.thelancet.com accessed November 2014. 10.1016/S0140-6736(02)08357-5 11978358

[41] Hejazi SB, Medeiros S, Mohammadi R, Lin J, Dalal K (2013). Patterns of intimate partner violence: a study of female victims in Malawi. J Inj Violence Res.

